# McMaster Optimal Aging Portal: an evidence-based database for geriatrics-focused health professionals

**DOI:** 10.1186/s13104-017-2595-8

**Published:** 2017-07-11

**Authors:** Angela M. Barbara, Maureen Dobbins, R. Brian Haynes, Alfonso Iorio, John N. Lavis, Parminder Raina, Anthony J. Levinson

**Affiliations:** 1 Health Information Research Unit, Department of Health Research Methods, Evidence and Impact, McMaster University, 1280 Main Street West, Hamilton, ON L8S 4K1 Canada; 2 School of Nursing, McMaster University, Hamilton, Canada; 3 McMaster Health Forum, Centre for Health Economics and Policy Analysis, Department of Health Research Methods, Evidence and Impact, McMaster University, Hamilton, Canada; 4 Department of Political Science, McMaster University, Hamilton, Canada; 5 Department of Global Health and Population, Harvard T.H. Chan School of Public Health, Boston, Massachusetts USA; 6 Canadian Longitudinal Study on Aging, Department of Health Research Methods, Evidence and Impact, McMaster University, Hamilton, Canada; 7 Division of e-Learning Innovation, McMaster University, Hamilton, Canada

**Keywords:** Healthy aging, Online health information, Bibliographic database, Evidence-based medicine

## Abstract

**Objective:**

The objective of this work was to provide easy access to reliable health information based on good quality research that will help health care professionals to learn what works best for seniors to stay as healthy as possible, manage health conditions and build supportive health systems. This will help meet the demands of our aging population that clinicians provide high quality care for older adults, that public health professionals deliver disease prevention and health promotion strategies across the life span, and that policymakers address the economic and social need to create a robust health system and a healthy society for all ages.

**Results:**

The McMaster Optimal Aging Portal’s (Portal) professional bibliographic database contains high quality scientific evidence about optimal aging specifically targeted to clinicians, public health professionals and policymakers. The database content comes from three information services: McMaster Premium LiteratUre Service (MacPLUS™), Health Evidence™ and Health Systems Evidence. The Portal is continually updated, freely accessible online, easily searchable, and provides email-based alerts when new records are added. The database is being continually assessed for value, usability and use. A number of improvements are planned, including French language translation of content, increased linkages between related records within the Portal database, and inclusion of additional types of content. While this article focuses on the professional database, the Portal also houses resources for patients, caregivers and the general public, which may also be of interest to geriatric practitioners and researchers.

## Introduction

As the population in many countries steadily ages and individuals get older [1, 2], there is also a societal shift in perceptions about aging. Rather than associating aging with physical and mental decline, older individuals are expecting to stay healthy, active and engaged for as long as possible [3]. There is also the recognition that resources should be dedicated to support older persons to stay as healthy as possible and connected to their communities [3].

The shift in population demographics has implications for professionals working in the health care field. While geriatricians and other clinicians will be required to continue providing high quality care for unhealthy older adults, there is a growing expectation for public health professionals to deliver disease prevention and health promotion strategies focused on older adults and for policymakers to address the economic and social need to create a healthy society for all ages [4]. Easy access to reliable health information based on good quality research will help health professionals and policymakers to stay abreast of what works best to stay healthy, manage health conditions and build supportive health systems.

There is widespread acceptance that health care practices and policies should be based on research evidence [5]. However, the translation of research evidence into practice remains a challenge due to barriers at different levels of health care [6]. At the innovation level, these barriers include feasibility, credibility, and accessibility. At the individual professional level, lack of awareness, time constraints, lack of knowledge (or skills in research methods), and information overload are some of the barriers [7]. Necessary, but not sufficient, conditions for closing this “evidence-practice gap” include information support services, evidence retrieval systems, improved dissemination of research, and access to applicable, regularly updated, pre-appraised synopses of the evidence [6, 8–10]. This indicates a need for a database or resource from which high quality geriatric health evidence could be retrieved for policy, public health, and clinical care decisions.

## The McMaster Optimal Aging Portal

### Rationale

The McMaster Optimal Aging Portal’s professional bibliographic database contains high quality scientific evidence about optimal aging specifically targeted to clinicians, public health professionals and policymakers. The search engine allows users to quickly examine synthesized evidence on the available research, be alerted to new evidence as it is published, and get direct access to updated information that can be used to help geriatric patients, communities and jurisdictions to be as healthy as possible. The database is freely accessible via the McMaster Optimal Aging Portal (Portal), which was officially launched on October 1, 2014. The Portal also houses resources for patients, caregivers and the general public, which may also be of interest to geriatric practitioners and researchers, and is described elsewhere [11]. This article focuses on the Portal professional database.

### Database construction and content

The Portal database content comes from three information services: McMaster Premium LiteratUre Service (MacPLUS™) [12–14], Health Evidence™ [15] and Health Systems Evidence [16–18]. The features of each repository are summarized in Table 1.

**Table 1:** Features of the three health information services that provide evidence to the Portal’s professional database

Feature	McMasterPLUS™	Health Evidence™	Health Systems Evidence
Focus	Clinical	Public health	Health system arrangements and implementation strategies
Types of documents	Overviews of systematic reviews; Systematic reviews; Meta-analyses; Original articles	Overviews of systematic reviews; Systematic reviews; Meta-analyses	Evidence briefs for policy; Overviews of systematic reviews; Systematic reviews; Systematic reviews in progress (protocols); Systematic reviews being planned (registered review titled); Economic evaluations/costing studies; Health reform descriptions; Health system descriptions; Canada’s health systems documents (available only in Canada unless subscribed); Ontario’s health system documents (available only in Ontario unless subscribed); Intergovernmental organizations’ health systems documents
Inclusion criteria	Must meet explicit criteria for scientific merit for the prediction, diagnosis, prognosis, prevention, treatment, or economics of a health problem and have a minimum mean score of 4/7 for clinical relevance plus minimum mean score of 4/7 for newsworthiness	5 criteria for relevance: Systematic review, Relevant to public health or health promotion practice, Subject is effectiveness, Includes evidence on outcomes, Describes search strategy	2 overarching criteria: Relevant to health system governance, financial or delivery arrangements, or implementation strategies; Meets the individual criteria for one of the document types
Quality raters	Physicians from each pertinent discipline (61 possible clinical disciplines in total, e.g., internal medicine, cardiology, psychiatry)	Health Evidence™ reviewers	McMaster Health Forum staff
Quality rating tool	McMaster Online Rating of Evidence (MORE) system [13]; Clinician comments	Health Evidence™ Quality Assessment Tool	Assessing the Methodological Quality of Systematic Reviews (AMSTAR) [18]
Quality ratings	Relevance: 7 = Direct and highly relevant, 6 = Definitely relevant, 5 = Probably relevant, 4 = Possibly relevant-likely of indirect or peripheral relevance at best; Newsworthiness: 7 = Useful information, most practitioners in my discipline definitely don’t know this, 6 = Useful information, most practitioners in my discipline probably don’t know this, 5 = Useful information, most practitioners in my discipline possibly don’t know this, 4 = Useful information, most practitioners in my discipline possibly already know this	8–10/10 = strong; 5–7/10 = moderate; 1–4/10 = weak	Score out of 11 (or lower if some criteria are not applicable)
Database fields	Title; Authors; Full journal citation; PubMed-indexed for MEDLINE (PMID); Link to user-friendly summary; Link to the abstract in PubMed; Quality ratings; Scientific abstract; Clinical comments provided by raters from each relevant discipline	Title; Quality rating; Full journal citation; Links, if available, to: – Article full-text, – Abstract in PubMed, – Related podcast, – User-friendly summary or Cochrane plain language summary, – Related webinar; Scientific abstract; Keywords	Title; Findings: – Links to user-friendly summary, if available, – Links to scientific abstract or document summary, – Link to full-text report, if available for free; Recency, quality and context of findings: – Year published, – Quality rating, – Country focus; Additional details about the research: – Type of document, – Type of question, – Focus, e.g., specific, general, – Priority area, if relevant, – Target, e.g., country, – Health systems topic(s), – Theme, e.g., optimal aging, health promotion/primary prevention, – Domain, Publication details: – Full citation, – Author email, – Digital object identifier (DOI)
Indexing	Medical subject headings (MeSH); Systematized Nomenclature of Medicine—Clinical Terms (SNOMED—CT)	Health Evidence Keywording Tool™	Health Systems Evidence taxonomy of health system governance, financial and delivery arrangements and implementation strategies that can support change in health systems
Search filters	Discipline; Category: – Primary prevention/health promotion, – Treatment, – Quality improvement, – Diagnosis, – Prognosis, – Etiology, – Economics, – Clinical prediction guide	Topic area (Interests); Setting; Intervention strategy; Intervention delivery method; Published date; Review quality rating; Review type; Text options; Primary prevention/health promotion	Type of document; Topic; Domain; Low- and middle-income country focus; Publication date; Focus; Primary prevention/health promotion
Email alert fields	Disciplines: – Emergency medicine, – General practice/family practice, – Occupational and environmental health, – Public health, – General internal medicine—primary care (United States), – Hospital doctor/hospitalists, – Internal medicine (or subspecialties), – Gynecology, – Psychiatry, – Anesthesiology, – Surgery—general (or subspecialties), – Special interest—pain; Cut-off scores: – Relevance: 4–7, – Newsworthiness: 4–7; Alert frequency: – Daily, – Every other day, – Every 3 days, – Every 4 days, – Every 5 days, – Every 6 days, – Weekly	Interests: – Addiction/substance abuse (or subcategories, – Chronic diseases (or subcategories), – Communicable disease/infection, – Dental health, – Emergency preparedness and response, – Environmental health, – Health through the ages (or subcategories), – Injury prevention/safety, – Mental health, – Nutrition, – Physical activity, – Sexual health, – Social determinants of health: Alert frequency: monthly	Health system topics: – Delivery arrangement, – Financial arrangement, – Governance arrangement, – Implementation strategy; Priority domains: – Diseases or: • Infectious diseases, • Non-communicable diseases, • Other (or subcategories); – Providers or: • Allied health professional. • Informal/family caregivers, • Lay/community health workers, • Nurse, • Pharmacist, • Physician (or specialty), – Sectors or: • Home care, • Hospital care, • Long-term care, • Primary care, • Public health, • Rehabilitation, – Technologies or: • Devices, • Diagnostics, • Drugs, • Surgery; Alert frequency: monthly

MacPLUS™ was created in 1991 and continuously updated since. The intellectual property for the process of critical appraisal and clinical ratings belongs to McMaster University, a not-for-profit, public university in Hamilton, Ontario, Canada. MacPLUS™ is supported by research funds and licensing contracts with academic, professional and commercial publishers which collectively cover the production costs and research dedicated to improving the service [12–14]. Development of Health Evidence™ began in 2000 and was officially launched in 2005. The service was initially supported by the Canadian Institute of Health Research (CIHR) and is sustained through a number of funded projects [15, 19–21]. Health Systems Evidence is an initiative of the McMaster Health Forum and was developed with contributions from a number of professional groups [17]. The service is supported by a collaboration between McMaster Health Forum’s Impact Lab and Cochrane Canada (https://www.healthsystemsevidence.org/about). To be included in the Portal database, the content from each service is filtered for its application to adults 60 years of age and older. The database is continually being expanded with new records/publications, and includes original studies and systematic reviews and select single studies (e.g., high quality single studies for MacPLUS™ and economic evaluations for Health Systems Evidence) with older adults and research on health promotion, disease prevention or the management of health conditions. As of September 15, 2015, the Portal database contained 29,247 bibliographic records (26,116 clinical records, 1357 public health records and 1774 policymaker records).

### Interface development

We used HTML5, CSS3, and Javascript jQuery components incorporating, but not limited to, KendoUI. We leveraged the extensibility of the Telerik Sitefinity ASP.NET web content management system to enhance the existing components/widgets, and create our own model-view-controller (MVC)-based ones. Communication between the presentation layer of the website and the database is handled in the backend using a custom C# application programming interface (API) to query the remote databases; and is delivered locally through a RESTful API to the site through the ServiceStack platform. This allows us to prevent cross-origin JavaScript object notation (JSON) issues, and restrict access using the native permissions in the web content management system.

The system is built on the Microsoft ASP.NET 4.5 framework, using a SQL 2008 R2 database for both the evidence articles and web content management system. We also use a social integration Software-as-a-Service model to ensure all pages, posts, resources and links are shareable; as well as incorporating a Twitter widget on the site, where appropriate.

The Sitefinity content management system’s multilingual content and translation management features (http://www.sitefinity.com/multilingual-content) were used, aiming for a fully bilingual site in English and French. This multilingual framework provides the potential for additional languages in the future.

### User interface

**Fig. 1:**
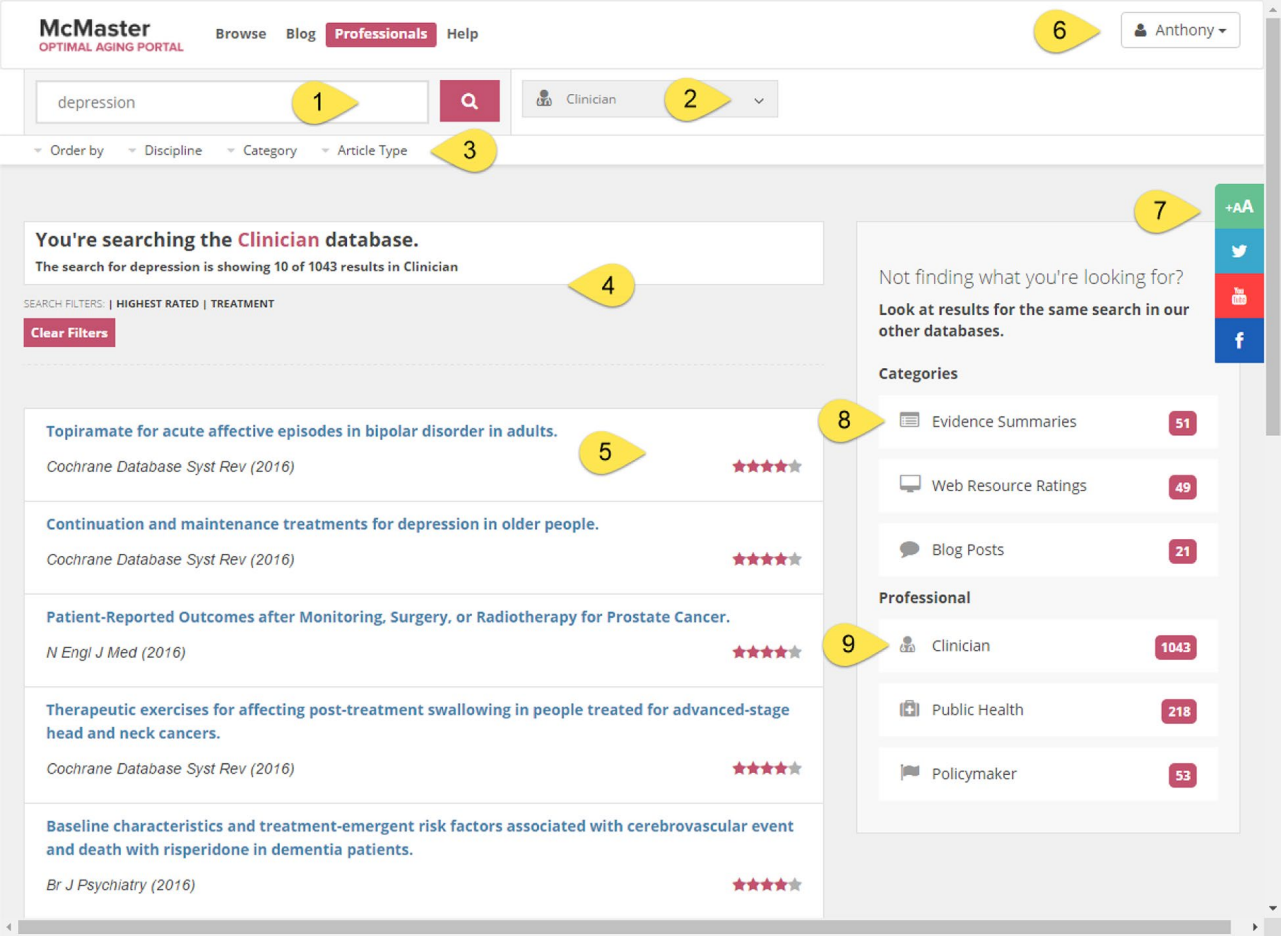
Results for search of the professional database. *1* Main search box. *2* Dropdown menu to select professional database. *3* Sorting and filtering options. *4* Number of search results. *5* Rating for the professional record. *6* Dropdown menu for profile (showing user name). *7* Buttons for adjusting font size and posting to Twitter, Youtube and Facebook. *8* Results for each type of “citizen” (non-professional) record. *9* Results for each type of professional record

**Fig. 2:**
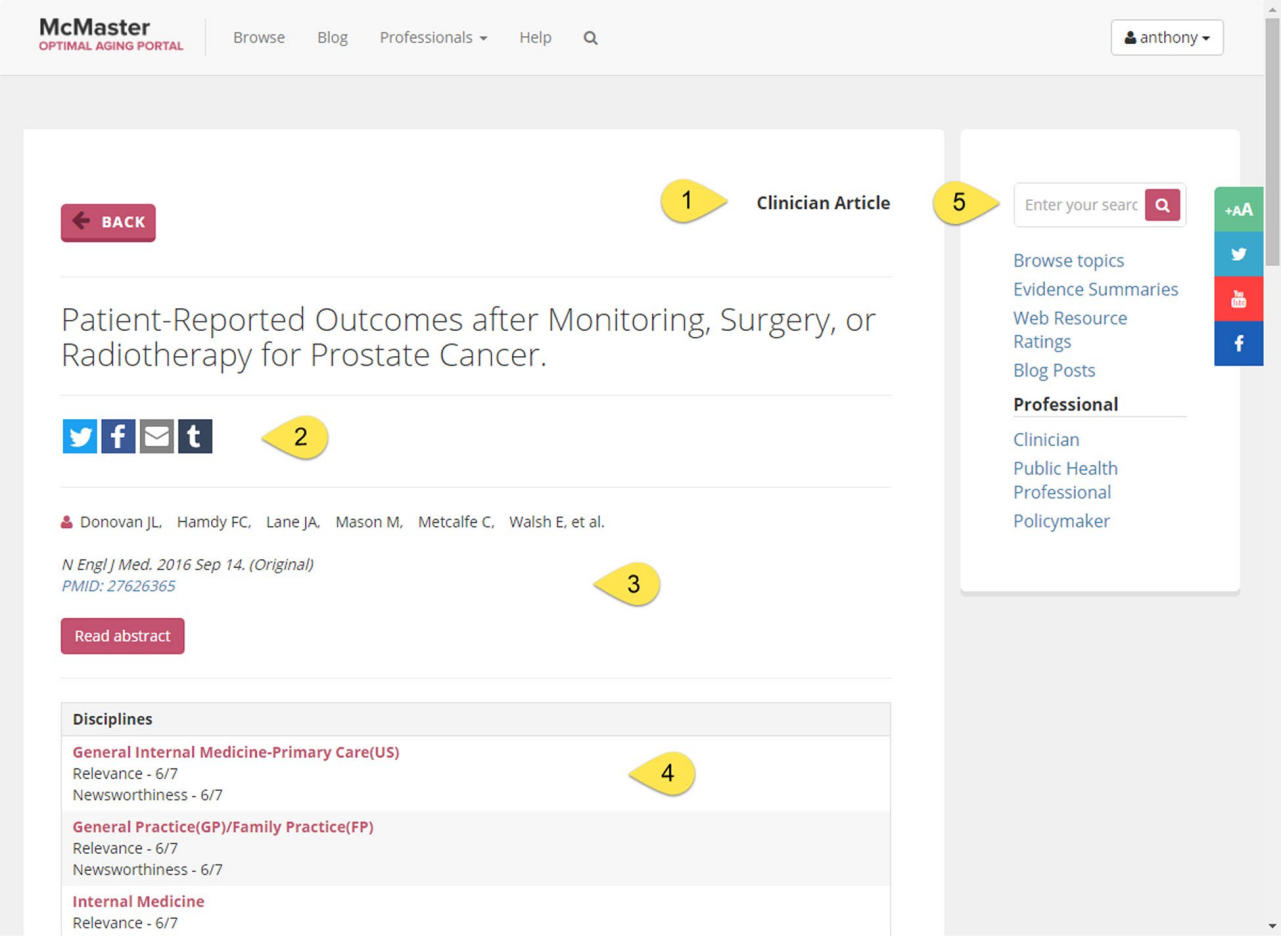
Clinical record on the Portal. *1* Type of article. *2* Buttons for sharing content via social media or email. *3* Journal citation. *4* Rating by clinical discipline. *5* Main search box

To house the database on the Portal, we used an iterative website design and development approach using “agile software development” methods (http://en.wikipedia.org/wiki/Agile_software_development). This approach encompasses the overall design, including the website interface, architecture, registration functionality and database integration. The Portal is built using a mobile first approach to ensure a stable responsive design that scales to any size phone, tablet or desktop (Figs. 1, 2).

The user experience is optimized by developing a user profile and logging into one’s profile while on the site. Visitors are prompted to identify themselves as either clinician, public health professional, policymaker (or general public/citizen, which is not discussed in this article). The Portal database was developed with a primary goal of easy navigation and searchability. The results that best match the users’ identified professional role are displayed most prominently in the central portion of the webpage. The relevant content from the different disciplinary services is also retrieved following the search. The related records from the other professional (and citizen) content available on the Portal are available on the right side of the page (Fig. 1). For all search results, the number of items retrieved per professional (and citizen) content type is posted, and all search results in each category are ordered by evidence rating rank. In short, the multidisciplinary content is retrieved following each search.

The content of the Portal database is not static; rather it is constantly updated. The Portal provides an easy way to access high quality, rated research evidence from the huge and ever growing health sciences literature. The information contained in the database can provide support for evidence-based practice, evidence-based public health, and evidence-informed policy. It can facilitate a number of research endeavors such as bibliometric, scientometric and knowledge-based informatics research, user and role search preferences, and information needs analysis.

The database was pilot-tested with 20 professional users (7 clinicians, 9 public health workers, and 4 policymakers) and eight non-professional users. Overall, the Portal was perceived as a useful resource for professionals. Based on specific feedback, we implemented a number of changes to improve display, usability, functionality, navigation, and address any technical glitches.

### Comparison with similar existing databases

While other bibliographic databases exist, the Portal database bring together a wide range of features by including all of the characteristics listed in Table 2. Few bibliographic databases bring together all of these features. For example, the Cochrane Database of Systematic Reviews contains high quality systematic reviews in healthcare. However, this database contains only reviews published specifically for the Cochrane Collaboration [22]. The Portal database includes all systematic reviews, meta-analyses and research articles that meet our criteria.

**Table 2:** Database characteristics of the Portal

Multidisciplinary content (medicine/nursing/rehabilitation, public health, policy) in one location
Synthesized evidence (with some single studies for clinicians and policymakers)
Content is independently rated for quality
Focus on geriatrics, including health aging and disease prevention
Content is constantly updated
Easily searchable and search results are organized by discipline (McMasterPLUS), practice area (Health Evidence™) or health system topics (Health Systems Evidence)
Freely accessible
Links to patient resources

**Fig. 3:**
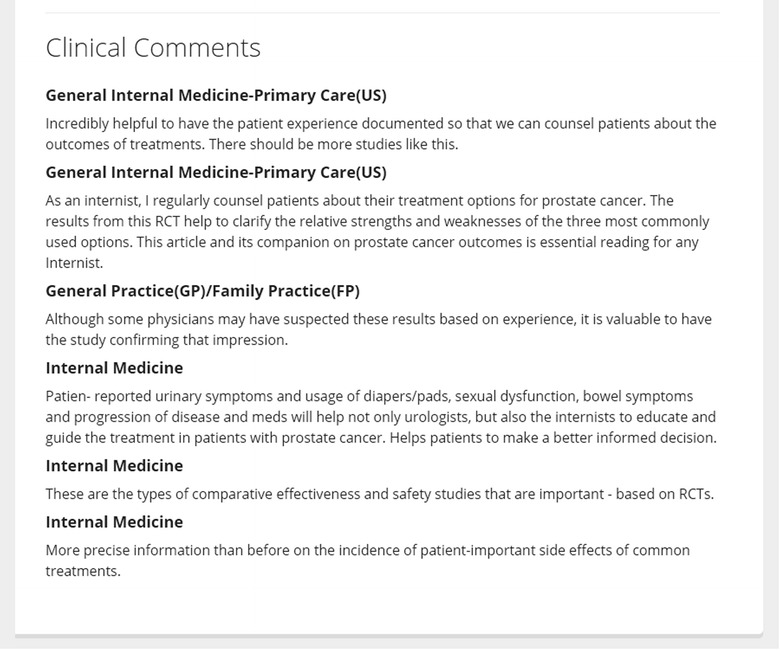
Commentary on a clinical record in the Portal

The Portal content has been independently rated for quality by MacPLUS™, Health Evidence or Health Systems Evidence before being included in the database (Table 1), with additional commentary from various types of clinicians for clinical articles (Fig. 3). This is not the case with most medical or scholarly databases. Access to the Portal database does not require a paid subscription, as needed for *Global Health* (https://www.ebscohost.com/academic/global-health) and UpToDate (http://www.uptodate.com).

### Planned development of new features

We strive to continue improving the value and usability of the Portal’s database content for professionals. A number of improvements are planned for the near future (Table 3).

**Table 3:** Planned improvements

Plan	Reason	Evaluation
Increased support for French language/translated content in the database	Canada is an officially bilingual country, and we want to provide access, where possible, to French content	Usage statistics; Web analytics; Satisfaction surveys
Integration with clinical systems	Suggested by professional users, members of the Portal Expert Advisory Committee; aligns with the desire to have resources that are useful and efficient at clinical point of care. Clinician content has been indexed with SNOMED-CT; however, additional resources are required to complete	Formative evaluation
Increased linkages between related records	Improve navigation using shared indexing/taxonomies; e.g., linking professional record to content for citizens	Web analytics
Improved ‘social sharing’ of database records	Suggested by actual users and the Portal marketing team. Implemented using commercial Software-as-a-Service social sharing tools, e.g., AddThis and/or custom programming	Sharing analytics
More granular alerts and personalization by specific topics and sub-topics	Suggested by actual users and the clinicians on the Portal development team. Additional resources are required to implement	Formative evaluation; Needs assessment
Further user testing with target audience to improve usability	On-going activity, as part of continuous quality improvement approach	User testing; Satisfaction surveys
Information retrieval analysis to investigate the search behavior of professional users and improve search engine functionality and effectiveness	Suggested by Portal development team	Feasibility analysis
Addition of other types of records, such as social science-related evidence and decision aids for clinicians to use with patients	Suggested by the Portal Steering Committee and Expert Advisory Committee	Focus groups; User testing; Satisfaction surveys

### Potential use cases

We present fictional case studies to demonstrate three possible uses of the Portal database. The individuals depicted below are not real people; rather, they were invented for illustrative purposes only. The case studies are based on a combination of use cases with scenarios and personas that were created during the development and testing phases [23], feedback from pilot testing with professional users, and subsequent feedback from actual users regarding how they were using the Portal database.

#### Case study 1—Geriatrician

**Fig. 4:**
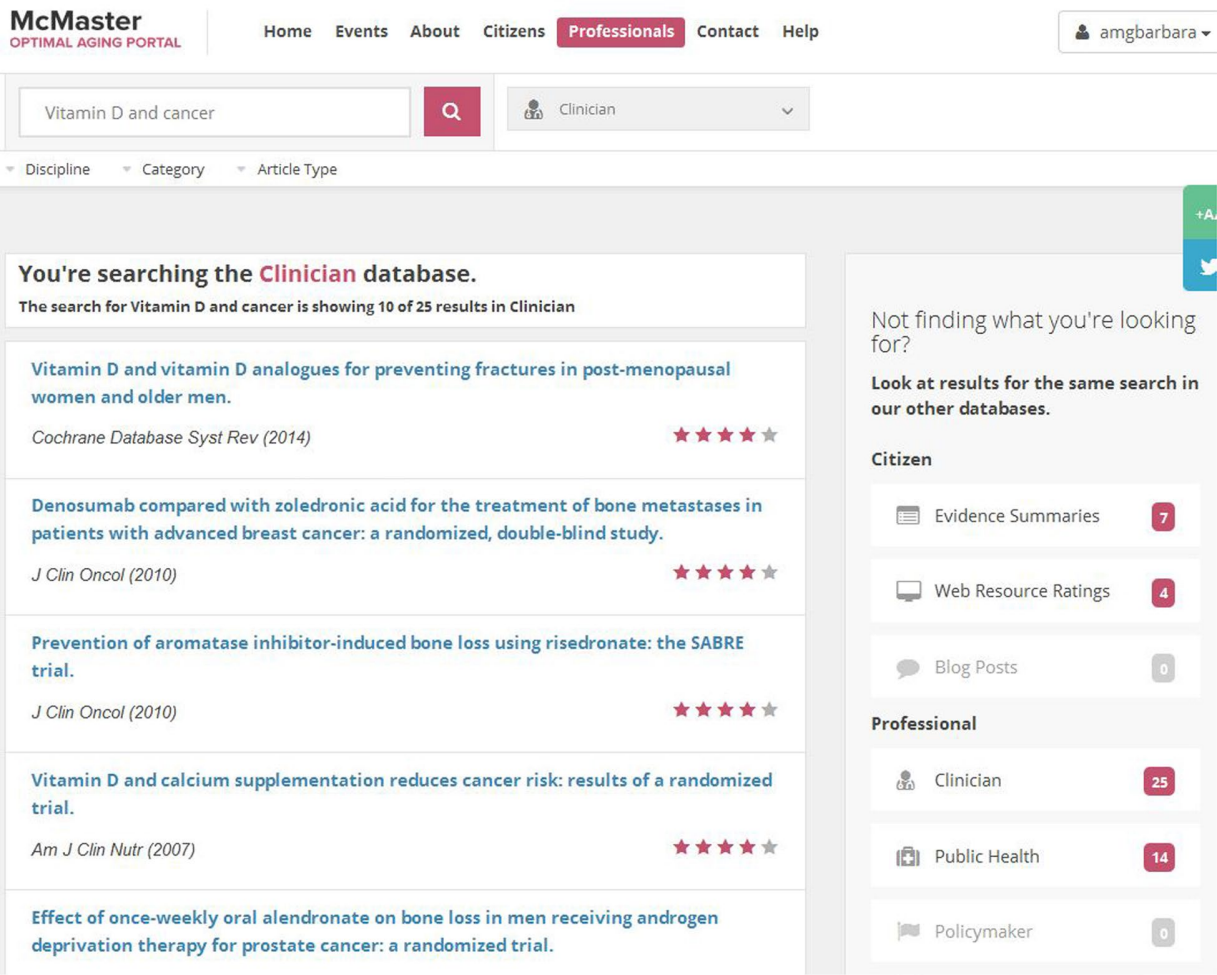
Results for the search “vitamin D and cancer”

Dr. Seow is visited by an 80-year old healthy patient who mentions a study that appeared on the evening news and attracted her attention. The study claimed that vitamin D can prevent cancer. However, her friend was taking vitamins and she was only 70 years old when she died of cancer. The patient is wondering whether she should start taking vitamin D. The physician logs on to the Portal using the clinic computer. By searching for “vitamin D and cancer” he finds 25 high quality systematic reviews in the clinician service on the topic (Fig. 4). He narrows the results to five by selecting the “primary prevention” filter for “Category”. During the visit, he reviews the results of the structured abstracts available for the five relevant systematic reviews and concludes that he will not recommend vitamin D supplementation to his patient for cancer prevention. On the search results page, the physician sees that seven patient-friendly Evidence Summaries are available. He reviews the titles with the patient and they choose the summary titled “Combined vitamin D and calcium supplementation can reduce fracture risk but not necessarily cancer risk” to print and review together. The rating of 5 out of 5 stars is a high quality rating, which assures both Dr. Seow and the patient that the information is reliable. The patient has her tablet with her so the physician shows her how to access the Portal herself. Later, the patient reviews the Evidence Summaries with her daughter and decides that, although there is no clear relationship between vitamin D and cancer prevention, she will start taking vitamin D plus calcium for bone health and prevention of fractures.

#### Case study 2—Public health professional

Diana is developing a new initiative on injury and falls prevention. She is interested in what the evidence says about community-wide falls prevention strategies. A search on “fall prevention” brings up 77 public health articles. To be certain that she reviews the best quality evidence, she selects “Strong” under the “Review quality rating” filter which reduces the results set to 40. She directs a member of her team to review the 40 articles, while she also examines the clinician and policymaker records. Based on a number of high quality systematic reviews, the team decides to start a program offering group exercise sessions and instructional training for home exercises focused on improving balance.

#### Case study 3—Policymaker

The news has recently been focusing on patient safety and adverse drug reactions in long-term care/nursing homes. Joseph, a policymaker, wants to know where to invest time and resources. He logs onto the Portal to look for a systematic review on the topic. A search for “long term care” retrieves 30 policymaker records. Joseph is pleased to find a 2015 article entitled “Computerised clinical decision support systems to improve medication safety in long-term care homes: A systematic review.” He is able to link to the freely available full-text report, and shares the structured abstract with his colleagues. At the next team meeting, they discuss the finding of the systematic review; i.e., that computerized clinical decision support systems may improve the quality of prescribing decisions in long-term care, better detect potential adverse drug reactions, and show potential to reduce injury risk among older adults. Together, they plan to implement a pilot study in collaboration with a long-term care facility. Joseph also signs up to receive monthly email alerts containing links to newly identified research evidence on the topic of delivery arrangements and home care and long-term care sectors.

## Conclusions

The Portal database puts in *one place* the *highest quality available* research evidence on geriatrics. By having a single evidence database to access, professionals no longer have to remember which database to go to depending on their query and do not have to visit individual databases for different professional perspectives on a health topic. Health professionals and researchers can access research evidence about healthy aging that has been synthesized on a single topic. The database contains only research evidence in which users can have confidence or it provides a quality rating that allows users to adjust their confidence level.

## Limitations

The limitations of the Portal professional database include those related to the individual methodology of the three information sources (Table 1). Only specific types of content will be included, such as systematic reviews; whereas, qualitative research will not be represented. There also exists some heterogeneity between groups, i.e., quality rating tools used. MacPLUS™ employs reviewers from different clinical disciplines; whereas, Health Evidence™ and Health Systems Evidence do not involve content experts. Some health professionals and researchers may be interested in a wider scope of research, i.e., the entire population unrestricted to age, for which they will have to access MacPLUS™, Health Evidence™ or Health Systems Evidence directly.

## Abbreviations

AMSTAR: Assessing the Methodological Quality of Systematic Reviews
API: application programming interface
CIHR: Canadian Institute of Health Research
DOI: digital object identifier
JSON: JavaScript object notation
MESH: medical subject headings
MORE: McMaster Online Rating of Evidence
MVC: model-view-controller
PLUS: Premium LiteratUre Service
PMID: PubMed-indexed for MEDLINE
SNOMED—CT: Systematized Nomenclature of Medicine—Clinical Terms
